# Analysis of all non-fatal self-harm cases in an urban area of Japan during pre- and peri-pandemic periods of COVID-19: a population-based study

**DOI:** 10.1265/ehpm.23-00143

**Published:** 2023-11-01

**Authors:** Takashi Yamauchi, Koga Hashimoto, Takashi Shimazaki, Machi Suka, Tadashi Takeshima

**Affiliations:** 1 Department of Public Health and Environmental Medicine, The Jikei University School of Medicine, 3-25-8 Nishi-shimbashi, Minato-ku, Tokyo 105-8461, Japan; 2 Kawasaki City Inclusive Rehabilitation Center, 5-1 Nisshin-cho, Kawasaki-ku, Kawasaki-shi, Kanagawa 210-0024, Japan

**Keywords:** Non-fatal self-harm, COVID-19, Pre-hospital medical record, Incidence rate ratio

## Abstract

**Background:**

This study aimed to examine population-based characteristics of non-fatal self-harm in an urban area during pre- and peri-pandemic periods of COVID-19 by sex, age, and severity of self-harm, using pre-hospital medical emergency records.

**Methods:**

We used a registry of all pre-hospital medical records of self-harm cases that occurred in Kawasaki City, Japan, between January 2018 and December 2021. Adjusted incidence rate ratios (IRRs) with 95% confidence intervals (CIs) were calculated using Poisson regression models with the log-transformed population by year, sex, age group, and ward as an offset term.

**Results:**

During the 4-year study period, 1,534 patients were transported by ambulance due to non-fatal self-harm and were alive on arrival at the hospital. Among women, the number of non-fatal self-harm cases increased by 1.2-fold in 2021 compared with that in 2018. The incidence rate of “severe” non-fatal self-harm among men aged 19 years or younger in 2021 (IRR 4.82, 95% CI 1.25–18.65) and that among women aged 50–59 years in 2020 (IRR 2.51, 95% CI 1.06–5.95) significantly increased compared with that 2018 and 2019. The incidence rate of “mild” self-harm among women aged 20–29 years tended to be higher in 2021 than in 2018 and 2019 (IRR 1.42, 95% CI 0.95–2.12, P = 0.085).

**Conclusions:**

During the peri-pandemic period of COVID-19, the incidence rate of “severe” non-fatal self-harm among men aged 19 years or younger and women aged 50–59 years, as well as that of “mild” self-harm among women aged 20–29 years, sharply increased compared with that during the pre-pandemic period. Our findings suggest that in urban areas during public health crises such as a pandemic, it is important to take measures to reduce the risk of non-fatal self-harm in young women, in addition to strengthening counseling and support for young women at risk for completed suicide.

**Supplementary information:**

The online version contains supplementary material available at https://doi.org/10.1265/ehpm.23-00143.

## Background

Data from the World Health Organization show that Japan’s suicide mortality rate is among the highest in the world [1]. The government implemented national suicide prevention strategies (i.e., the General Principles of Suicide Prevention Policy) in 2007, highlighting the importance of aftercare for people who committed “non-fatal” self-harm, defined as self-injurious behaviors with the intent to die or with unclear/unknown intentions.

Studies of suicidal behavior, primarily in North America and Europe, have shown that a previous history of non-fatal, deliberate self-harm is a strong risk factor for completed suicide [2–9]. Thus, examining the characteristics of people who attempted non-fatal self-harm (e.g., tendency to attempt self-harm using less lethal methods than those used by suicide completers) may contribute to preventing subsequent severe suicide attempts, including those which result in death. While population-based studies on completed suicides (i.e., “fatal” self-harm) using vital statistics in each country [1] and hospital-based studies on attempted suicides [5, 10–12] have been reported worldwide, detailed population-based statistics on non-fatal self-harm have not been published in Japan.

During the peri-pandemic period of novel coronavirus disease 2019 (COVID-19), in high/upper-middle-income countries such as European and North American countries, the total number of suicide deaths has remained largely unchanged or declined in the early stage of the pandemic compared with the pre-pandemic period [13]. On the other hand, among young people in Japan, suicide mortality rates have been on the rise, and these increases remained stable during the pandemic [14, 15]. Similarly, during the pandemic, the number of suicide deaths among Japanese women has increased beyond the expected number of deaths, regardless of employment status or age [16]. Thus, it is possible that the risk for committing non-fatal self-harm as well as fatal self-harm among women, especially young women, has increased during the pandemic. However, while several population-based studies have reported on the state of non-fatal self-harm during the pandemic in Western countries [17–19], very few population-based studies have been conducted on incidence rates of non-fatal self-harm in Asian countries, especially in Far East countries such as Japan, despite the relatively high suicide rates in those countries [1].

To our knowledge, only one population-based study has examined changes in incidence rates of ambulance transport due to fatal/non-fatal self-harm in an urban area in Japan between pre- and peri-COVID-19 pandemic periods. Nakao et al. reported that among those aged 20–29 years living in an urban area in western Japan, the incidence rates of ambulance transport due to self-harm significantly increased in 2020 compared with that in 2016 [20]. However, that study did not consider the intensity/severity of non-fatal self-harm (i.e., mild, moderate, and severe), and included only data from 2020 on self-harm during the peri-pandemic period. While there may be common and similar background factors between “severe” non-fatal self-harm and completed suicide cases, “mild” non-fatal self-harm cases may include those who injured themselves without the intention of committing suicide.

The present study aimed to examine population-based characteristics of all non-fatal self-harm patients transported by ambulance during the pre- and peri-pandemic periods of COVID-19 by sex, age, and severity of self-harm, using pre-hospital medical emergency records in a large city of the Greater Tokyo Area of Japan. Understanding the state of non-fatal self-harm, one of the strongest risk factors for completed suicide [1], in an urban area during the pandemic may contribute to preventing suicidal behaviors during the ongoing and future public health crises, and results of this study may offer useful implications for future suicide prevention policies and measures.

## Materials and methods

### Study design and population

The present study is a population-based observational study using a registry of all pre-hospital medical emergency records of self-harm cases that occurred in Kawasaki City between January 2018 and December 2021; in all cases, patients were transported by Fire Department ambulance. Kawasaki City is located in the Greater Tokyo Area, with a 2021 population of approximately 1.5 million residents. The Fire Department provided the anonymous database in September 2022.

Emergency records were completed by emergency medical service personnel and entered into the registry of the Fire Department. “Self-harm” cases were defined as those who injured themselves deliberately and identified by emergency workers based on an interview with the patient herself/himself and on-site observation.

Between January 2018 and December 2021, 1,812 self-harm patients were transported by ambulance to a medical institution. The inclusion criterion for the present study was patients living in Kawasaki City at the time of the occurrence of self-harm. Given that the objective of the present study was to examine the state of *non-fatal* self-harm among the general population, patients who were dead at the scene of self-harm or on arrival at the hospital (i.e., *fatal* self-harm cases) were excluded.

The study protocol was approved by the institutional review board of the Jikei University School of Medicine (No. 34-301(11454)).

### Variables

Emergency medical service workers collected information on each self-harm case using the standardized pre-hospital medical emergency record form. The information provided to the authors included the year of occurrence of self-harm, severity of non-fatal self-harm (“mild,” “moderate,” and “severe”), methods of self-harm, place of occurrence of self-harm (“residence” or “other”), and demographic variables such as sex, age, employment status (“employed,” “unemployed,” “student,” and “unknown”), and ward (i.e., resident area). The degree of severity of non-fatal self-harm was determined for each patient by a physician at the time of transport to the emergency department of a medical institution. Due to the difficulties of classifying the methods of self-harm registered in pre-hospital medical emergency records, we used the following three major categories: “self-cutting,” “self-poisoning,” and “other.” Details of “self-poisoning,” such as the type/name of toxic substances (e.g., prescription drugs, over-the-counter drugs), were not registered in pre-hospital medical emergency records.

### Statistical analysis

First, cross-tabulation was conducted between study year and demographic variables and severity of non-fatal self-harm. To compare the incidence rates of ambulance transport due to non-fatal self-harm in 2020 and 2021 with that in 2018 and 2019 (i.e., the pre-pandemic period), we calculated adjusted incidence rate ratios (IRRs) with 95% confidence intervals (CIs) using Poisson regression models with year, sex, age group, and ward (i.e., resident area) as independent variables and the log-transformed population by year, sex, age group, and ward as an offset term. We calculated sex-, age-, and severity-stratified IRRs for ambulance transport due to non-fatal self-harm to identify specific groups that were vulnerable to self-harm during the COVID-19 pandemic.

P < 0.05 was considered statistically significant. All analyses were performed using SPSS version 25 (IBM, Chicago, IL, USA).

## Results

**Fig. 1:**
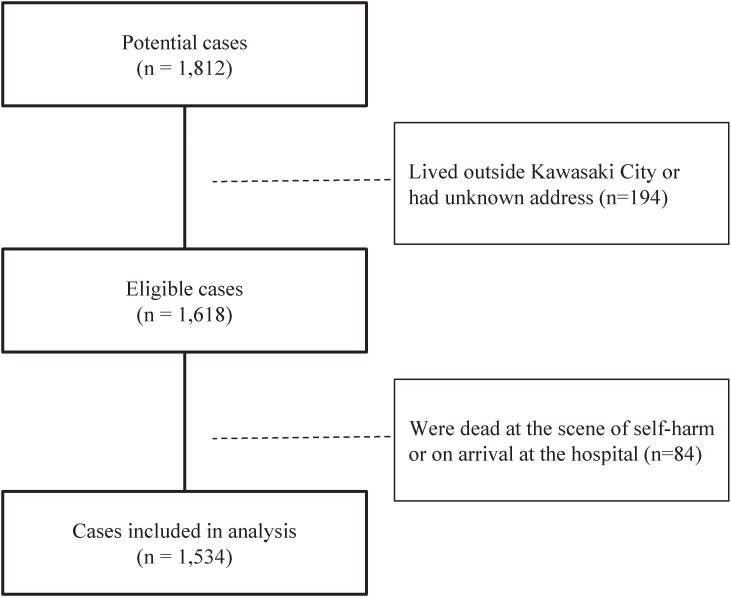
Flowchart of study sample selection

Between January 2018 and December 2021, there were a total of 1,812 self-harm cases in which patients were transported by ambulance to a medical institution. Of these, 194 patients who lived outside Kawasaki City at the time of ambulance transport and those with an unknown address or residual status were excluded. Among the 1,618 eligible patients, 84 who were dead at the scene or on arrival at the hospital were excluded, and the remaining 1,534 were included in the final analyses (Fig. 1).

Tables 1-1 and 1-2 and Appendix 1 summarize the characteristics of non-fatal self-harm patients in Kawasaki City by year, sex, and ward. In total, 458 men (29.9%) and 1,076 women (70.1%) were transported to the emergency department of a medical institution due to non-fatal self-harm. Among women, the number of non-fatal self-harm cases (n = 295) increased by 1.2-fold in 2021 compared with that in 2018 (n = 245). Regardless of year, “severe” non-fatal self-harm was more frequent among men, whereas “mild” and “moderate” self-harm was more frequent among women. Among women, regardless of year, the number of non-fatal self-harm cases was higher in the 20–29 year age group than in other age groups. Regardless of year, self-poisoning was the most common method of non-fatal self-harm, especially among women. The distribution of incidence rate of self-harm patients transported by ambulance per 100,000 by ward differed substantially in both sexes, especially in women (Appendix 1).

**Table 1-1:** Number of self-harm patients transported by ambulance by year (men)

	**Self-harm cases (n = 458)**							

	**2018** **(n = 109)**		**2019** **(n = 122)**		**2020** **(n = 118)**		**2021** **(n = 109)**	
			
	**n**	**(%)**	**n**	**(%)**	**n**	**(%)**	**n**	**(%)**
Severity								
Severe	38	(34.9)	44	(36.1)	53	(44.9)	46	(42.2)
Moderate	39	(35.8)	43	(35.2)	37	(31.4)	38	(34.9)
Mild	32	(29.4)	35	(28.7)	28	(23.7)	25	(22.9)
Age (years)								
<20	7	(6.4)	8	(6.6)	7	(5.9)	13	(11.9)
20–29	11	(10.1)	22	(18.0)	22	(18.6)	19	(17.4)
30–39	19	(17.4)	18	(14.8)	17	(14.4)	23	(21.1)
40–49	28	(25.7)	27	(22.1)	29	(24.6)	16	(14.7)
50–59	17	(15.6)	25	(20.5)	22	(18.6)	18	(16.5)
>59	27	(24.8)	22	(18.0)	21	(17.8)	20	(18.3)
Methods								
Self-cutting	17	(15.6)	20	(16.4)	9	(7.6)	11	(10.1)
Self-poisoning	43	(39.4)	44	(36.1)	42	(35.6)	47	(43.1)
Other	49	(45.0)	58	(47.5)	67	(56.8)	51	(46.8)
Place of self-harm								
Residence	85	(78.0)	105	(86.1)	104	(88.1)	85	(78.0)
Other	24	(22.0)	17	(13.9)	14	(11.9)	24	(22.0)
Employment status								
Employed	39	(35.8)	43	(35.2)	42	(35.6)	38	(34.9)
Unemployed	50	(45.9)	68	(55.7)	55	(46.6)	55	(50.5)
Student	8	(7.3)	7	(5.7)	9	(7.6)	11	(10.1)
Unknown	12	(11.0)	4	(3.3)	12	(10.2)	5	(4.6)

**Table 1-2:** Number of self-harm patients transported by ambulance by year (women)

	**Self-harm cases (n = 1,076)**							

	**2018** **(n = 245)**		**2019** **(n = 282)**		**2020** **(n = 254)**		**2021** **(n = 295)**	
			
	**n**	**(%)**	**n**	**(%)**	**n**	**(%)**	**n**	**(%)**
Severity								
Severe	43	(17.6)	69	(24.5)	63	(24.8)	65	(22.0)
Moderate	131	(53.5)	126	(44.7)	115	(45.3)	124	(42.0)
Mild	71	(29.0)	87	(30.9)	76	(29.9)	106	(35.9)
Age (years)								
<20	25	(10.2)	25	(8.9)	27	(10.6)	32	(10.8)
20–29	72	(29.4)	93	(33.0)	77	(30.3)	107	(36.3)
30–39	39	(15.9)	59	(20.9)	43	(16.9)	44	(14.9)
40–49	39	(15.9)	49	(17.4)	41	(16.1)	43	(14.6)
50–59	32	(13.1)	17	(6.0)	30	(11.8)	32	(10.8)
>59	38	(15.5)	39	(13.8)	36	(14.2)	37	(12.5)
Methods								
Self-cutting	30	(12.2)	34	(12.1)	38	(15.0)	35	(11.9)
Self-poisoning	156	(63.7)	174	(61.7)	156	(61.4)	193	(65.4)
Other	59	(24.1)	74	(26.2)	60	(23.6)	67	(22.7)
Place of self-harm								
Residence	221	(90.2)	254	(90.1)	231	(90.9)	260	(88.1)
Other	24	(9.8)	28	(9.9)	23	(9.1)	35	(11.9)
Employment status								
Employed	36	(14.7)	65	(23.0)	57	(22.4)	65	(22.0)
Unemployed	167	(68.2)	158	(56.0)	149	(58.7)	161	(54.6)
Student	27	(11.0)	36	(12.8)	29	(11.4)	42	(14.2)
Unknown	15	(6.1)	23	(8.2)	19	(7.5)	27	(9.2)

Table 2 shows the IRRs of non-fatal self-harm by severity, year, and sex using Poisson regression models with years 2018 and 2019 as the reference category. During the peri-pandemic period, among women, the incidence rate of “mild” self-harm was significantly higher in 2021 than in 2018 and 2019 (IRR 1.31, 95% CI 1.03–1.68).

**Table 2:** Incidence rate ratios (IRRs) of self-harm cases transport by severity, year, and sex

**Severity**	**Year**	**Men**		**Women**	
	
		**Adjusted ** **IRR**	**(95% CI)**	**Adjusted ** **IRR**	**(95% CI)**
**Total**					
	2021	0.94	(0.75–1.18)	1.10	(0.95–1.27)
	2020	1.01	(0.81–1.27)	0.95	(0.82–1.10)
	2018/2019	1.00	(Reference)	1.00	(Reference)
**Severe**					
	2021	1.11	(0.77–1.59)	1.15	(0.85–1.56)
	2020	1.28	(0.91–1.81)	1.11	(0.82–1.51)
	2018/2019	1.00	(Reference)	1.00	(Reference)
**Moderate**					
	2021	0.92	(0.63–1.35)	0.95	(0.76–1.17)
	2020	0.90	(0.61–1.32)	0.88	(0.71–1.09)
	2018/2019	1.00	(Reference)	1.00	(Reference)
**Mild**					
	2021	0.74	(0.47–1.17)	1.31*	(1.03–1.68)
	2020	0.83	(0.53–1.29)	0.94	(0.72–1.24)
	2018/2019	1.00	(Reference)	1.00	(Reference)

Incidence rate ratios were adjusted for age and ward.

* P < 0.05.

Table 3 shows the IRRs of non-fatal self-harm by age group, year, and sex using Poisson regression models. During the peri-pandemic period, the incidence rate of non-fatal self-harm among women aged 20–29 years tended to be higher in 2021 than in 2018 and 2019 (IRR 1.23, 95% CI 0.96–1.57, P = 0.099).

**Table 3:** Incidence rate ratios (IRRs) of self-harm cases by age, year, and sex

**Age group**	**Year**	**Men**		**Women**	
	
		**Adjusted IRR**	**(95% CI)**	**Adjusted IRR**	**(95% CI)**
**<20 years**					
	2021	1.78	(0.85–3.74)	1.32	(0.85–2.06)
	2020	0.94	(0.38–2.31)	1.10	(0.69–1.75)
	2018/2019	1.00	(Reference)	1.00	(Reference)
**20–29 years**					
	2021	1.13	(0.64–1.98)	1.23	(0.96–1.57)
	2020	1.32	(0.77–2.26)	0.89	(0.68–1.17)
	2018/2019	1.00	(Reference)	1.00	(Reference)
**30–39 years**					
	2021	1.27	(0.76–2.14)	0.92	(0.64–1.31)
	2020	0.93	(0.52–1.65)	0.88	(0.62–1.27)
	2018/2019	1.00	(Reference)	1.00	(Reference)
**40–49 years**					
	2021	0.60	(0.35–1.05)	1.02	(0.71–1.47)
	2020	1.08	(0.68–1.67)	0.95	(0.66–1.38)
	2018/2019	1.00	(Reference)	1.00	(Reference)
**50–59 years**					
	2021	0.78	(0.45–1.36)	1.18	(0.76–1.84)
	2020	0.99	(0.59–1.65)	1.15	(0.73–1.81)
	2018/2019	1.00	(Reference)	1.00	(Reference)
**>59 years**					
	2021	0.80	(0.48–1.35)	0.94	(0.63–1.39)
	2020	0.85	(0.51–1.42)	0.92	(0.62–1.37)
	2018/2019	1.00	(Reference)	1.00	(Reference)

Incidence rate ratios were adjusted for ward.

Tables 4-1 to 4-3 show the IRRs of non-fatal self-harm by age group, year, sex, and severity using Poisson regression models. During the peri-pandemic period, among women aged 50–59 years, the incidence rate of “severe” self-harm was significantly higher in 2020 than in 2018 and 2019 (IRR 2.51, 95% CI 1.06–5.95); among men aged 19 years or younger, the incidence rate of “severe” self-harm was significantly higher in 2021 than in 2018 and 2019 (IRR 4.82, 95% CI 1.25–18.65). Among women aged 20–29 years, the incidence rate of “mild” self-harm tended to be higher in 2021 than in 2018 and 2019 (IRR 1.42, 95% CI 0.95–2.12, P = 0.085).

**Table 4-1:** Incidence rate ratios (IRRs) of severe self-harm cases by age, year, and sex

**Age group**	**Year**	**Men**		**Women**	
	
		**Adjusted IRR**	**(95% CI)**	**Adjusted IRR**	**(95% CI)**
**<20 years**					
	2021	4.82*	(1.25–18.65)	1.52	(0.61–3.78)
	2020	2.03	(0.41–10.08)	1.12	(0.41–3.02)
	2018/2019	1.00	(Reference)	1.00	(Reference)
**20–29 years**					
	2021	1.98	(0.69–5.63)	1.00	(0.48–2.06)
	2020	1.42	(0.45–4.47)	1.01	(0.49–2.09)
	2018/2019	1.00	(Reference)	1.00	(Reference)
**30–39 years**					
	2021	2.51	(1.00–6.05)	1.15	(0.60–2.21)
	2020	1.35	(0.48–3.78)	0.65	(0.29–1.43)
	2018/2019	1.00	(Reference)	1.00	(Reference)
**40–49 years**					
	2021	0.48	(0.20–1.16)	0.91	(0.43–1.91)
	2020	0.85	(0.42–1.73)	1.42	(0.75–2.68)
	2018/2019	1.00	(Reference)	1.00	(Reference)
**50–59 years**					
	2021	0.68	(0.27–1.75)	2.01	(0.82–4.94)
	2020	1.77	(0.87–3.58)	2.51*	(1.06–5.95)
	2018/2019	1.00	(Reference)	1.00	(Reference)
**>59 years**					
	2021	0.84	(0.38–1.83)	1.02	(0.51–2.05)
	2020	1.23	(0.62–2.45)	0.86	(0.41–1.81)
	2018/2019	1.00	(Reference)	1.00	(Reference)

Incidence rate ratios were adjusted for ward.

* P < 0.05.

**Table 4-2:** Incidence rate ratios (IRRs) of moderate self-harm cases by age, year, and sex

**Age group**	**Year**	**Men**		**Women**	
	
		**Adjusted IRR**	**(95% CI)**	**Adjusted IRR**	**(95% CI)**
**<20 years**					
	2021	2.57	(0.69–9.59)	1.12	(0.57–2.20)
	2020	1.01	(0.19–5.52)	0.76	(0.35–1.64)
	2018/2019	1.0	(Reference)	1.0	(Reference)
**20–29 years**					
	2021	1.14	(0.45–2.90)	1.16	(0.83–1.63)
	2020	1.81	(0.80–4.11)	0.87	(0.60–1.27)
	2018/2019	1.0	(Reference)	1.0	(Reference)
**30–39 years**					
	2021	1.02	(0.41–2.52)	0.58	(0.31–1.07)
	2020	0.58	(0.19–1.75)	0.83	(0.49–1.42)
	2018/2019	1.0	(Reference)	1.0	(Reference)
**40–49 years**					
	2021	0.84	(0.37–1.90)	1.05	(0.61–1.79)
	2020	1.22	(0.60–2.50)	1.02	(0.60–1.75)
	2018/2019	1.0	(Reference)	1.0	(Reference)
**50–59 years**					
	2021	0.57	(0.21–1.55)	0.90	(0.45–1.81)
	2020	0.59	(0.22–1.60)	1.02	(0.52–2.00)
	2018/2019	1.0	(Reference)	1.0	(Reference)
**>59 years**					
	2021	0.73	(0.29–1.87)	0.67	(0.35–1.29)
	2020	0.37	(0.11–1.27)	0.79	(0.42–1.46)
	2018/2019	1.0	(Reference)	1.0	(Reference)

Incidence rate ratios were adjusted for ward.

**Table 4-3:** Incidence rate ratios (IRRs) of mild self-harm cases by age, year, and sex

**Age group**	**Year**	**Men**		**Women**	
	
		**Adjusted IRR**	**(95% CI)**	**Adjusted IRR**	**(95% CI)**
**<20 years**					
	2021	0.25	(0.10–2.03)	1.50	(0.69–3.27)
	2020	0.50	(0.11–2.36)	1.61	(0.76–3.45)
	2018/2019	1.00	(Reference)	1.00	(Reference)
**20–29 years**					
	2021	0.69	(0.25–1.93)	1.42	(0.95–2.12)
	2020	0.85	(0.32–2.21)	0.89	(0.60–1.42)
	2018/2019	1.00	(Reference)	1.00	(Reference)
**30–39 years**					
	2021	0.73	(0.26–2.03)	1.28	(0.70–2.35)
	2020	1.01	(0.41–2.51)	1.19	(0.64–2.21)
	2018/2019	1.00	(Reference)	1.00	(Reference)
**40–49 years**					
	2021	0.46	(0.10–2.12)	1.09	(0.56–2.14)
	2020	1.34	(0.48–3.78)	0.41	(0.16–1.07)
	2018/2019	1.00	(Reference)	1.00	(Reference)
**50–59 years**					
	2021	1.28	(0.49–3.36)	1.13	(0.51–2.49)
	2020	0.38	(0.10–1.73)	0.59	(0.22–1.60)
	2018/2019	1.00	(Reference)	1.00	(Reference)
**>59 years**					
	2021	0.83	(0.29–2.34)	1.33	(0.66–2.69)
	2020	0.83	(0.29–2.37)	1.2	(0.60–2.56)
	2018/2019	1.00	(Reference)	1.00	(Reference)

Incidence rate ratios were adjusted for ward.

## Discussion

The present study revealed changes in trends of incidence rates of non-fatal self-harm resulting in ambulance transport in Kawasaki City from 2018 to 2021 (i.e., pre- and peri-pandemic periods of COVID-19) by severity using a registry of pre-hospital medical emergency records. There were a total of 1,534 cases in which patients were transported by ambulance due to non-fatal self-harm in Kawasaki City during the study period, with 70% occurring in women. During the peri-pandemic period, a non-significant increasing trend (1.4-fold, p < 0.1) in the incidence rate of “mild” self-harm among women aged 20–29 years was observed in 2021 compared with that in 2018 and 2019.

Consistent with previous studies that analyzed pre-hospital emergency records in Japan during the peri-pandemic period [20], most non-fatal self-harm cases identified in emergency records in Kawasaki City occurred among women during the peri-pandemic period. The incidence rate of self-harm per 100,000 by ward also differed substantially for both sexes, especially women (Appendix 1). While the reason for this difference in the incidence rate of self-harm between wards is unclear, relatively higher rates among women in Kawasaki ward might reflect higher suicide rates among women in this ward [21]; therefore, we calculated the IRRs with 95% CIs using Poisson regression models with year, sex, age group and ward as independent variables in the present study. Moreover, self-poisoning and self-cutting were the most frequent methods of non-fatal self-harm for both men and women, consistent with a previous report [22]. High-lethality methods such as hanging and jumping from a high place were less frequent among non-fatal self-harm cases, especially among female cases. Therefore, preventive efforts should focus on people at high risk for self-harm primarily using drugs or sharp objects.

For women, the incidence rate of “severe” self-harm among those aged 50–59 years in 2020 was significantly higher than that in 2018 and 2019, although the estimation range of 95% CIs was poor. Unfortunately, annual nationwide age-specific statistics on self-harm have not been reported in Japan. In terms of suicide deaths in Japan, for both sexes, while the number of suicide deaths and suicide mortality rates consistently declined between 2011 and 2019, these indicators increased in 2020 [14, 15]. By age group, suicide rates among women aged 50–59 years increased from 11.5 per 100,000 people in 2019 to 12.7 in 2020; this was the first increase in suicide rates compared with the previous year among women aged 50–59 since 2011 [15]. Given that “severe” self-harm and completed suicide likely share common background factors, such as the reason(s) for those behaviors, and that approximately 25% of suicide completers in Kawasaki City appeared to have had a history of suicide attempts in each year of the study period [23], the increase in suicide deaths among women aged 50–59 years in 2020 might reflect a similar trend observed in the present study regarding the incidence rate of “severe” self-harm in this particular group in 2020.

The incidence rate of “severe” self-harm among men aged 19 years or younger in 2021 was significantly higher than that in 2018. In Japan, among students, suicide mortality rates have been on the rise, and these increases remained stable even during the COVID-19 pandemic [14, 15]. During the height of the COVID-19 pandemic, opportunities for students to communicate with each other decreased due to the closing of schools/universities and the implementation of remote teaching and e-learning systems, and consequently, students may have experienced an increased level of psychological distress [24] and lower engagement with learning [25]. This vulnerability to suicidal behaviors might explain the sharp increase in the incidence rate of “severe” self-harm in this particular group in 2021, although the estimation of 95% CIs was poor due to the small number of “severe” self-harm cases in the present study.

Among women, the number of non-fatal self-harm cases increased by 1.2-fold in 2021 compared with that in 2018. Previous studies on self-harm in European countries have reported that the occurrence of self-harm increased during the peri-pandemic period of COVID-19 [26, 27]. In addition, as shown in Tables 1-2 and 3, the number and IRR of non-fatal self-harm among women aged 20–29 years showed increasing trends in 2021 compared with 2018 and 2019. These findings are in line with a report that incidence rates of ambulance transport due to self-harm among those aged 20–29 years increased during the peri-pandemic period, according to a previous population-based study using ambulance transport records in a metropolitan area in western Japan [20]. Similarly, the Ministry of Health, Labour and Welfare and the Fire and Disaster Management Agency of Japan reported that the total number of patients transported by ambulance due to self-harm in Japan increased after 2020, although those reports did not include information on the state of self-harm by sex [15]. Thus, increased incidence rates of ambulance transport due to non-fatal self-harm, especially among women living in urban areas, might be observed generally across Japan during the peri-pandemic period.

The present study also found that, during the peri-pandemic period, the incidence rate of “mild” self-harm among women aged 20–29 years showed an increasing trend (1.4-fold) in 2021 compared with that in 2018 and 2019. On the other hand, the number of suicide deaths among women aged 20–29 years in Kawasaki City remained at the same level during the 4-year study period (i.e., 10 in 2018, 13 in 2019, 12 in 2020, and 9 in 2021) [21]. These findings imply that, during the height of the COVID-19 pandemic, the quarantine policy and community containment measures might have led to a population-level increase in psychological distress among young women living in urban areas, leading to the increase in non-fatal self-harm. The Japanese government declared a state of emergency regarding COVID-19 for the first time in April 2020 and three times thereafter until the end of December 2021 due to rapid increases in the number of reported cases of COVID-19. As suggested in a previous study [20], women aged 20–29 years may be more vulnerable to changes in political, socio-economic, and personal life situations experienced during the COVID-19 pandemic, such as the quarantine policy, community containment measures, and a decrease in direct communication with the widespread application of telework/telecommuting and e-learning systems, although strict social distancing measures (e.g., lockdown) for the COVID-19 pandemic had not been imposed on the public in Japan as of December 2021. Therefore, they may have experienced psychological distress due to fear of getting sick and, especially, issues related to finances. As suggested in previous studies on self-harm and suicidal behaviors during the peri-pandemic period [20, 28], promoting access to mental health care and enhancing financial and social support could contribute to a reduction in the incidence of non-fatal self-harm among women under public health crises.

### Strengths and limitations

The present population-based study using pre-hospital emergency records allowed us to analyze all non-fatal self-harm cases that occurred in a region (i.e., Kawasaki City) during a specific time period, i.e., the ongoing peri-pandemic period of COVID-19. To our knowledge, this is the first study to examine population-based characteristics of non-fatal self-harm in an urban area during the peri-pandemic period of COVID-19 by severity of self-harm, using pre-hospital medical emergency records.

The present study also has some limitations worth noting. First, pre-hospital emergency records did not include detailed information on long-term outcomes following hospital admission due to self-harm. In addition, the degree of severity of non-fatal self-harm might have been misclassified since it was determined for each patient by a physician at the time of transport to the emergency department of a medical institution. Thus, the use of pre-hospital emergency records might have introduced some information bias. Second, as suggested in a previous study analyzing a population-based data in Japan [20], we could not separate non-suicidal self-harm cases from suicidal self-harm cases due to the lack of relevant information in pre-hospital emergency records. Moreover, since the diagnosis of “self-harm” was made by emergency medical service workers based on on-site observations and an interview at the scene of the occurrence of self-harm within a limited period of time. These factors could result in an underestimation/overestimation of the incidence of non-fatal self-harm in the present study. However, regarding the severity of self-harm, Yamada et al. [11] clarified characteristics of suicide attempters transported to a critical emergency unit, revealing very few differences between high-lethality and low-lethality groups in terms of employment status, current psychiatric treatment, history of deliberate self-harm, and family history of suicidal behavior. Third, we could not identify non-fatal self-harm cases with a history of repeated self-harm during the 4-year study period, since the registry of pre-hospital emergency records analyzed in this study did not include any personally identifiable information, such as name, birth date, and address. Finally, we restricted our analysis to Kawasaki City residents who attempted non-fatal self-harm from 2018 to 2021. Furthermore, unlike other countries, strict social distancing measures, including lockdown, were not introduced in Japan. Therefore, the applicability of the current study findings to a population with a different background should be carefully examined.

## Conclusions

During the peri-pandemic period of COVID-19, the incidence rate of “severe” non-fatal self-harm among men aged 19 years or younger and women aged 50–59 years, as well as that of “mild” self-harm among women aged 20–29 years, sharply increased compared with that during the pre-pandemic period. These findings suggest that in urban areas during public health crises such as a pandemic, it is important to take measures to reduce the risk of non-fatal self-harm in young women, in addition to strengthening counseling and support for young women who are at risk for completed suicide. Such measures may include training suicide prevention gatekeepers with an emphasis on supporting young women, and physical/financial/psychological support for young women in need (e.g., single mothers).

## Abbreviations

COVID-19: coronavirus disease 2019

## Supplementary information

**Additional file 1:** **Appendix 1.** Distribution of incidence rates of self-harm patients transported by ambulance per 100,000 by year, sex, and ward.

## Declarations

### Data availability

Data cannot be shared for privacy or ethical reasons.
