# Supplementary material for: Analysis of all non-fatal self-harm cases in an urban area of Japan during pre- and peri-pandemic periods of COVID-19: a population-based study
Source: Environ Health Prev Med. 2023 Nov 1;28:65. doi: 10.1265/ehpm.23-00143 (PMC10636291; doi:10.1265/ehpm.23-00143)
Supplement: Supplementary file 1 — Additional file 1: Appendix 1. Distribution of incidence rates of self-harm patients transported by ambulance per 100,000 by year, sex, and ward. [file ehpm-28-065-s001.docx]

| Appendix 1. Distribution of incidence rate of self-harm patients transported by ambulance per 100,000 by year, sex, and ward | | | | | | | | | |
| --- | --- | --- | --- | --- | --- | --- | --- | --- | --- |
|  | Men | | | |  | Women | | | |
|  | 2018 | 2019 | 2020 | 2021 |  | 2018 | 2019 | 2020 | 2021 |
| Ward |  |  |  |  |  |  |  |  |  |
| Kawasaki-ku | 16.9 | 17.6 | 13.6 | 23.3 |  | 45.8 | 54.7 | 52.8 | 46.7 |
| Saiwai-ku | 17.8 | 21.0 | 23.1 | 12.7 |  | 35.0 | 29.7 | 35.5 | 39.1 |
| Nakahara-ku | 10.6 | 8.3 | 14.2 | 9.0 |  | 15.8 | 28.8 | 25.4 | 39.8 |
| Takatsu-ku | 9.4 | 15.4 | 11.2 | 13.7 |  | 34.7 | 31.8 | 27.1 | 39.7 |
| Miyamae-ku | 15.9 | 17.6 | 11.5 | 10.6 |  | 33.9 | 32.0 | 24.1 | 19.9 |
| Tama-ku | 17.9 | 20.5 | 17.7 | 15.8 |  | 34.7 | 36.2 | 41.4 | 62.1 |
| Asao-ku | 11.5 | 11.5 | 18.3 | 12.6 |  | 32.6 | 50.7 | 30.0 | 22.4 |
